# A Systematic Review of MicroRNA in Glioblastoma Multiforme: Micro-modulators in the Mesenchymal Mode of Migration and Invasion

**DOI:** 10.1007/s12035-012-8349-7

**Published:** 2012-10-02

**Authors:** Heidi G. Møller, Andreas P. Rasmussen, Hjalte H. Andersen, Kasper B. Johnsen, Michael Henriksen, Meg Duroux

**Affiliations:** Department of Biomedicine, Institute for Medicine and Health Technology, Aalborg University, Fredrik Bajers Vej 3B, 9220 Aalborg Øst, Denmark

**Keywords:** MicroRNA, Glioblastoma, Glioma, Migration, Invasion, Mesenchymal mode of migration and invasion (MMMI), Systematic review, EMT

## Abstract

Glioblastoma multiforme (GBM) is an incurable form of brain cancer with a very poor prognosis. Because of its highly invasive nature, it is impossible to remove all tumor cells during surgical resection, making relapse inevitable. Further research into the regulatory mechanism underpinning GBM pathogenesis is therefore warranted, and over the past decade, there has been an increased focus on the functional role of microRNA (miRNA). This systematic review aims to present a comprehensive overview of all the available literature on the expression profiles and function of miRNA in GBM. Here, we have reviewed 163 papers and identified 253 upregulated, 95 downregulated, and 17 disputed miRNAs with respect to expression levels; 85 % of these miRNAs have not yet been functionally characterized. A focus in this study has been 26 interesting miRNAs involved in the mesenchymal mode of migration and invasion, demonstrating the importance of miRNAs in the context of the cellular niche. Both oncogenic and tumor-suppressive miRNAs were found to affect target genes involved in cell migration, cytoskeletal rearrangement, invasiveness, and angiogenesis. Clearly, the distinct functional properties of these miRNAs need further investigation and might hold a great potential in future molecular therapies targeting GBM.

**Electronic supplementary material:**

The online version of this article (doi:10.1007/s12035-012-8349-7) contains supplementary material, which is available to authorized users.

## Introduction

Glioblastoma multiforme (GBM) is a highly prevalent, incurable form of cancer emanating from the brain, accounting for 12–15 % of all brain tumors and approximately 70 % of all diagnosed gliomas [1, 2]. Among the WHO grades of astrocytoma, GBM is classified as being the most progressed and severe (grade IV). It is characterized by an extremely poor prognosis, reflected in a mean survival rate of only 3.3 % at 2 years and 1.2 % at 3 years [3, 4]. While GBM rarely metastasizes, it characteristically grows by infiltrating the surrounding brain tissue [5]. Because of this highly invasive nature, it is impossible to completely remove all tumor cells during surgical resection [6].

Today, treatment of GBM is primarily through tumor resection and subsequent radio- and chemotherapy, typically alkylating agents, e.g., temozolomide [7, 8]. Despite intensive efforts to improve current treatment and explore new therapeutic targets, pivotal clinical improvement has remained absent during the last decade [7, 9]. Other forms of treatment, such as photodynamic therapy, based on photooxidative reactions and accumulation of photo sensitizers in tumor tissue, can be used to facilitate tumor resection and target cell proliferation [10]. Molecular phenotyping of GBM is opening up the potential for molecularly targeted therapies. These can take the form of targeting specific components of oncogenic pathways, e.g., through delivery of a therapeutic gene or microRNA (miRNA) [9, 11–13]. Several miRNAs are differentially expressed in a variety of malignancies compared to corresponding healthy tissue. Some of these miRNAs have been shown to modulate oncogenes and tumor suppressors, as is the case for GBM. Therefore, miRNAs could hold a great potential in the future treatment of this disease.

## Biogenesis of miRNA

**Fig. 1:**
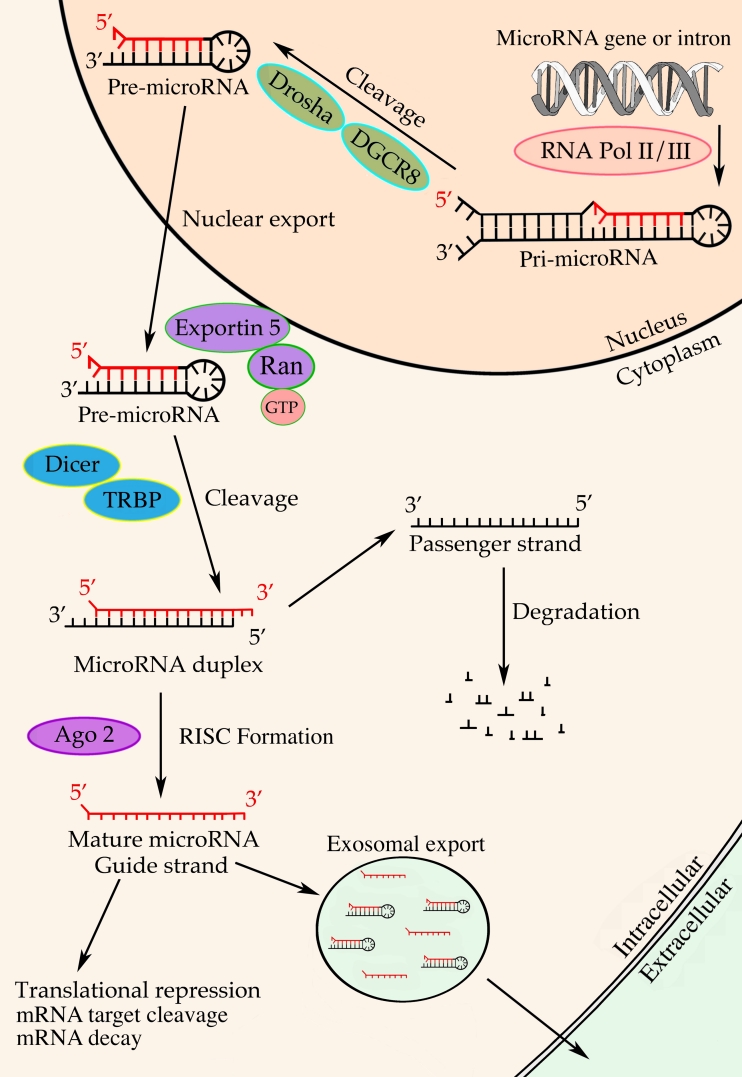
The linear biogenesis of miRNA. miRNA biogenesis involves transcription of pri-miRNA by RNA polymerase II/III, cleavage by the Drosha-DGCR8 complex to pre-miRNA, followed by export to the cytoplasm by Exportin-5 in the presence of Ran-GTP cofactor. In the cytoplasm, pre-miRNA is cleaved by the Dicer-TRBP complex to a miRNA duplex, which is unwound to a guide strand that is bound to Ago2 and incorporated into the RISC, and a passenger strand, which is degraded. Ultimately, miRNA binding to target mRNAs results in mRNA target cleavage, translational repression, or mRNA decay. A more novel fate of the miRNAs is the selective secretion via microvesicles or exosomes

In order to understand the context of miRNA in GBM pathology, we highlight here the essential steps in the biogenesis of miRNAs and the effects they exert on their downstream functional targets (Fig. 1). miRNAs are small RNA molecules of approximately 20–23 nucleotides, which have been identified as important regulators of mRNA translation [14]. The biogenesis of miRNA starts with transcription of miRNA genes by RNA polymerase II/III (Pol II/III), generating a primary transcript (pri-miRNA) that is both capped and polyadenylated. The transcript folds into a stem-loop structure via intramolecular base-pairing [14]. The stem-loop structure is cleaved to pre-miRNA by the Drosha/DGCR8 complex and actively transported out of the nucleus by Exportin-5 in the presence of Ran-GTP cofactor [15]. In the cytoplasm, the RNAseIII enzyme Dicer makes the final cleavage to a double-stranded miRNA, of which one strand is incorporated into the RNA-induced silencing complex (RISC), the cytoplasmic effector machine for miRNA. The other strand is degraded [16]. RISC is comprised of Dicer, the double-stranded RNA binding factor (TRBP) and Argonaut protein 2 (Ago2). The posttranscriptional RNA silencing is mediated through complementary binding of miRNA within RISC to the mRNA 3′ untranslated region, resulting in mRNA cleavage, translational inhibition, or mRNA decay [17]. This mRNA interference results in a decreased level of encoded proteins, hereby affecting an array of cellular processes, e.g., proliferation, migration, and apoptosis[18]. A new and less-studied fate of miRNAs is the selective excretion via lipoproteins or microvesicles, possibly functioning as a way of intercellular communication [19, 20].

## Studies of microRNA in Glioblastoma Multiforme

The scope of this paper is to provide the most up-to-date review of the current literature pertaining to miRNA studies in GBM. The first two papers investigating miRNA expression profiles in GBM were published almost simultaneously in 2005 [21, 22]. Ciafrè et al. demonstrated aberrant expression levels of numerous miRNAs when comparing GBM tumor samples to normal brain tissue [22]. While also analyzing miRNA expression levels, Chan et al. [21] were the first to investigate the functional properties of a single miRNA in GBM cell lines. They found that inhibition of miR-21 resulted in significantly increased apoptosis and, therefore, hypothesized that miR-21 could function as a micro-oncogene [21]. Since these early findings, the number of studies published on this subject has steadily increased, elucidating numerous interesting miRNA-mediated mechanisms in the tumorigenesis of GBM.

To obtain a comprehensive overview of the current knowledge on miRNA expression and function in GBM, all available literature on the subject was reviewed. A Medline database search on: “microRNA and Glioblastoma” plus “microRNA and Glioma”^1^ was performed (date of search entry: April 20, 2012). The results contained a total of 256 papers, of which 163 were found relevant, based on the title and abstract content. The 163 papers were reviewed, 102 of them met the inclusion criteria, which encompassed studies investigating expression levels of miRNAs in GBM tumor samples compared to normal brain tissue, and/or studies exploring the functional properties of selected miRNAs in GBM cell lines. Of the remaining 61 papers, 34 were studies primarily focusing on diagnostics, delivery, and prognostics and were categorized as editor’s notes or generally not the scope of the topic. Twenty-seven papers were reviews and review-like articles, discussing the role of miRNA in GBM. Clearly, none of the 27 reviews were highly systematic in their approach; primarily, they described few well-known and reviewed miRNAs. All relevant material extracted from the 102 papers that were reviewed has been presented in the form of a supplementary table (Supplementary Table 1). The content of this supplementary table provides the foundation for the material discussed in the following sections of the paper. Each section attempts to highlight the population of upregulated, downregulated, and novel miRNAs. Emphasis on the promising therapeutic potential of specific miRNAs is presented with a focus on the mesenchymal mode of migration and invasion (MMMI) in GBM.

## MicroRNAs Upregulated in Glioblastoma Multiforme

Based solely on the literature contained in this review, it appears that the most common dysregulation of miRNA in GBM is overexpression, thus, 256 miRNAs were found significantly overexpressed (Supplementary Table 1). Few of these, for example miR-17, miR-21, miR-93, and miR-221/222, have been intensively investigated with respect to both expression and functionality, but the functional properties of the vast majority remains completely unknown. Table 1 display all miRNAs that were found upregulated in greater than or equal to five studies and/or functionally investigated, with emphasis on luciferase-validated targets, in at least one study. In the following sections, miRNAs that are particularly interesting, extensively investigated, or novel are discussed.

**Table 1:** Upregulated miRNAs and their functional role in GBM

miRNA	Targets	Functional role when 1: overexpressed, 2: underexpressed	Reference
Hsa-mir-9	CAMTA1		[23, 24, 30–32]
Hsa-mir-9*	CAMTA1	2: Proliferation↓, Stemness↓	[25, 32]
Hsa-mir-10b^a^	HOXD10^d^	1:Invasiveness↑	[22–29]
		2: Invasiveness↓	
Hsa-mir-15b	CCNE1	1:Proliferation↑	[23–25, 31, 35, 100]
		2: Proliferation↓	
Hsa-mir-16			[24, 25, 30, 31, 36]
Hsa-mir-17^a^	POLD2, TGFβ-RII^b^, CTGF, CAMTA1	1:Angiogenesis↑, Growth↑	[24–26, 30–34]
		2: Viability↓, Apoptosis↑, Proliferation↓	
Hsa-mir-18a^a^	Smad4, CTGF	1:Angiogenesis↑, Growth↑	[25, 33, 34]
		2: Viability↓, Apoptosis↑, Proliferation↓	
Hsa-mir-20a^a^	TGFβ-RII^b^, CTGF	1:Angiogenesis↑, Growth↑	[25, 26, 30, 31, 33]
		2: Viability↓, Proliferation↓	
Hsa-mir-21^ac^	RECK^d^,TIMP3^d^, APAF1, ANP32A^d^, SMARCA4, Caspases, PTEN, Cdc25A, HNRPK, TAp63, Spry2^d^, LRRFIP1, PDCD4	1:Invasiveness↑	[21–28, 30, 31, 35–55]
		2: Invasiveness↓, Apoptosis↑, Viability↓, Proliferation↓, In vivo tumor volume↓, Chemosensitivity↑	
Hsa-mir-23a			[22–25, 35, 37, 52]
Hsa-mir-25^c^	Mdm2, TSC1	1:In vivo tumor volume↓	[22–26, 30, 101]
Hsa-mir-26a^c^	PTEN	1:In vivo tumor volume↑	[23, 25, 37]
Hsa-mir-27a	WEE1		[23, 25, 52]
Hsa-mir-30e^c^	IκBα^d^	1:Invasiveness↑, Proliferation↑, Angiogenesis↑, In vivo tumor volume↑	[25, 96]
		2: Invasiveness↓, Proliferation↓, Angiogenesis↓, In vivo tumor volume↓	
Hsa-mir-92 ^a^	CTGF	2: Viability↓, Proliferation↓	[25, 26, 33, 34]
Hsa-mir-93^ac^	Integrin-β8^d^	1:Angiogenesis↑, Proliferation↑, In vivo tumor volume↑	[23, 24, 26, 56]
Hsa-mir-106b			[23–26, 28, 52]
Hsa-mir-125b	Bmf	1:Invasiveness↑, Apoptosis↓, Proliferation↑	[25, 97, 102, 103]
Hsa-mir-146a^c^	Notch1	1:Proliferation↓, In vivo tumor volume↓, Migration↓	[24, 25, 104]
Hsa-mir-155		2: Viability↓, Apoptosis↑, Chemosensitivity↑	[24–26, 31, 36, 105]
Hsa-mir-182			[23–26, 37, 106]
Hsa-mir-183			[23, 25, 26, 106, 107]
Hsa-mir-210			[25, 26, 30, 31, 36]
Hsa-mir-221^c^	P27, Akt^d^, PUMA, P57, PTPμ^d^	1:Proliferation↑, Invasiveness↑, in vivo tumor volume↑, Apoptosis↓, Migration↑	[22, 35, 52, 53, 98, 108–113]
		2: Proliferation↓, Apoptosis↑, in vivo tumor volume↓	
Hsa-mir-222^c^	Akt, PUMA, P57, PTPμ^d^	1:Proliferation↑, Invasiveness↑, In vivo tumor volume↑, Apoptosis↓, Migration↑	[22, 35, 52, 98, 109–113]
		2: Proliferation↓, Apoptosis↑, in vivo tumor volume↓	
Hsa-mir-335^bc^	Daam1^d^	1:Viability↑, Invasiveness↑	[25, 59]
		2: Apoptosis↑, Invasiveness↓, In vivo tumor volume↓	
Hsa-mir-381^bc^	LRRC4	1:Proliferation↑, In vivo tumor volume↑	[60]
		2: Proliferation↓	

miRNAs consistently upregulated in glioblastoma. The table presents miRNAs with observed effects upon their downregulation and their validated targets. The cell lines in which the studies have been performed are presented. Only miRNAs that have been investigated in greater than or equal to five studies and/or have a validated target are included in this table. The cell lines applied in the annotated studies can be found in Supplementary Table 1

^a^Mentioned in the section “MicroRNAs Upregulated in Glioblastoma Multiforme”

^b^Mentioned in the section “MicroRNAs Upregulated in Glioblastoma Multiforme with Limited Functional Characterization”

^c^MicroRNAs whose down regulation is shown to inhibit tumour growth in vivo

^d^Targets involved in the mesenchymal mode of migration and invasion

### miR-10b

Since its first investigation by Ciafrè et al., overexpression of miR-10b in GBM has been confirmed in eight studies [22–29]. A significant WHO grade specific correlation of its overexpression has been observed, thus implying the pertinent role of miR-10b in GBM grade progression [28, 29]. Expression levels of urokinase receptor (uPAR) and Ras homolog gene family member C (RhoC) were found to be directly proportional to that of miR-10b, thereby enhancing the invasive capabilities of high-grade glioma [28]. Recently, a negative regulator of uPAR and RhoC, HOXD10, was confirmed as a direct target of miR-10b [29].

### miR-17 ~ 92-cluster

The expression of the miR-17 ∼ 92-cluster (comprising miR-17-3p, miR-17-5p, miR-18a, miR-19a, miR-19b, miR-20a, and miR-92a) is upregulated in GBM tumor samples and cell lines [24–26, 30–34]. The miR-17 ∼ 92-cluster has been shown to possess a variety of tumorigenic properties, mediated through direct targeting of antiproliferative genes (TGFBRII, SMAD4, and CAMTA1) and regulators of angiogenesis and DNA-repair (CTGF, and POLD2). Hence, inhibition of specific miR-17 ∼ 92-cluster members decreases viability and increases apoptosis in vitro [30, 33, 34].

### miR-21

Until now, the most extensively investigated miRNA is miR-21, which is consistently reported to be overexpressed in GBM in a WHO-grade specific manner [21–28, 30, 31, 35–55]. Forced downregulation of miR-21 reduces the oncogenic potential of GBM cell lines through inhibition of several cellular processes involved in malignancy maintenance. Proliferation is significantly reduced by cessation of miR-21 inhibition on the target genes ANP32A, SMARCA4, PTEN, SPRY2, and LRRFIP1 [35, 39, 46, 50]. This reduction is also associated with a decrease in the protein levels of key components of proliferation-linked signaling pathways, e.g., NF-κB and Ras [46]. These results were further substantiated by decreased tumor growth in immunodeficient mice [35, 43, 51]. miR-21 inhibition does also result in significantly increased levels of caspases, leading to an increase in apoptosis, mediated by decreased targeting of HNRPK, TAp63, and PDCD4 [40, 42, 51]. miR-21 affects invasion by decreasing the expression levels of RECK and TIMP3, which normally retains the levels of MMPs [38, 40]. Furthermore, transfection with antisense-miR-21 has been shown to significantly increase GBM cell line sensitivity to both radio- and chemotherapy [41, 44, 47, 48]. This aspect of its function makes miR-21 of particular interest for exploitation in molecular therapy [47].

### miR-93

While several studies have proven that miR-93 is upregulated in GBM [23, 24, 26], only one study has investigated its functional properties [56]. Fang et al. [57] used in vitro and in vivo studies to emphasize the functional role of this miRNA in angiogenesis. Using transfection, they further upregulated miR-93 in the model cell line U87. Following co-culture with endothelial cells, they observed increased endothelial cell proliferation and tube formation. Integrin-β8, which inhibits close association between tumor cells and endothelial cells, was found to be a direct target of miR-93. These results were then substantiated with in vivo assays, showing highly increased blood vessel formation in GBM xenograft tumors in mice, strongly validating miR-93 as an angiogenic inducer [56].

### MicroRNAs Upregulated in Glioblastoma Multiforme with Limited Functional Characterization

The case with many studies is that trends are followed around well-studied target genes in order to substantiate and validate results. To date, the center of attention has been pivotal around relatively few, well-investigated miRNAs, such as miR-15b, miR-21, miR-221, and miR-222 [58]. However, research on novel miRNAs, e.g., miR-335 and miR-381, has recently been published [59, 60]. miR-335 was upregulated in GBM and shown to increase proliferation and invasion in malignant astrocytoma, through direct targeting of DAAM1. These effects were reversed by miR-335 silencing [59, 61]. Another upregulated, novel miRNA, miR-381, was shown to increase the level of proliferation both in vitro and in xenograft mouse models. Through direct targeting of leucine-rich repeat C4 (LRRC4), miR-381 caused increased levels of phosphorylated MEK, ERK, and AKT, thus, impacting several important components of mitogenic signaling pathways [60]. Additionally, miR-16, miR-23a, miR-106b, miR-182, miR-183, and miR-210 were found to be consistently upregulated in greater than or equal to five studies but remain completely uninvestigated with respect to functional properties (Table 1).

## MicroRNAs Downregulated in Glioblastoma Multiforme

Throughout the reviewed literature, 95 miRNAs were reported as downregulated in GBM, compared to normal brain tissue (Supplementary Table 1). Of these, 28 were reported as downregulated in greater than or equal to five studies and/or functionally investigated, with emphasis on luciferase-validated targets, in at least one study (shown in Table 2). Interestingly, what one can infer is that miRNAs that are consistently downregulated in GBM often appear to possess anti-tumorigenic abilities (Table 2). Therefore, further investigation of these miRNAs could generate knowledge on valuable therapeutic targets. In the following section, downregulated, well-investigated miRNAs are highlighted. In conjunction with this, the novel miRNAs are discussed.

**Table 2:** Downregulated miRNA and their functional role in GBM

miRNA	Target	Functional role when overexpressed	Reference
Hsa-mir-7	FAK, EGFR, IRS2	Viability↓, Migration↓, Invasiveness↓, Proliferation↓, In vivo tumor volume↓, Radiosensitivity↓	[25, 27, 31, 62–65]
Hsa-mir-29b	PDPN^d^	Invasiveness↓ Proliferation↓ Apoptosis↑	[25, 36, 45, 114]
Hsa-mir-32^bc^	Mdm2, TSC1	In vivo tumor volume↓	[24, 45, 78]
Hsa-mir-34a^ac^	SIRT1^d^, c-Met, Notch1/2, PDGFRA^d,^ Msi1	Viability↓, Proliferation↓, Apoptosis↑, Invasiveness↓, In vivo tumor volume↓, Differentiation↑	[67–71]
Hsa-mir-100	ATM	Radiosensitivity↑	[115]
Hsa-mir-101^b^	EZH2 Msi1	Angiogenesis↓, Migration↓, Viability↓, Proliferation↓	[71, 79]
Hsa-mir-124	SNAI2^d^	Proliferation↓, Migration↓, Invasiveness↓, Stemness↓	[12, 24–27, 35–37, 45, 52, 116, 117]
Hsa-mir-125a		Invasiveness↓	[45, 114]
Hsa-mir-128^ac^	WEE1, p70S6K1, Msi1, E2F3a, Bmi-1, EGFR^d^, PDGFRA^d^	Angiogenesis↓, Proliferation↓, In vivo tumor volume↓	[22, 24–26, 31, 35, 37, 52, 55, 71–75]
Hsa-mir-128b	WEE1		[12, 22, 25, 26, 35, 37, 52]
Hsa-mir-129			[24–27, 101]
Hsa-mir-132			[12, 24–26, 31, 35, 52]
Hsa-mir-135a^c^	STAT6, Smad5, BMPR2	Inhibition causes: In vivo tumor volume↓, Apoptosis↑	[118]
Hsa-mir-137^ac^	CDK6, Msi1, Cox-2	Proliferation↓, Invasiveness↓, Migration↓, In vivo tumor volume↓	[25–27, 35, 45, 52, 71, 77]
Hsa-mir-138	Msi1	Proliferation↓	[24, 37, 71]
Hsa-mir-139-5p			[12, 25–27, 31]
Hsa-mir-146b-5p^c^	EGFR^d^	Invasiveness↓, Migration↓, Proliferation↓, In vivo tumor volume↓	[119]
Hsa-mir-149	RAP1B, Wnt-pathway	Proliferation↓, Migration↓	[31, 37]
Hsa-mir-153	Bcl-2, Mcl-1, Irs-2	Proliferation↓, Viability↓, Apoptosis↑	[26, 120]
Hsa-mir-181a	Bcl-2	Proliferation↓, Apoptosis↑, Invasiveness↓, Radiosensitivity↑	[22, 37, 121, 122]
Hsa-mir-181b		Proliferation↓, Apoptosis↑, Invasiveness↓	[22, 37, 45, 53, 121]
Hsa-mir-181d^c^	Bcl-2, K-Ras^d^	Proliferation↓, Apoptosis↑, In vivo tumor volume↓	[123]
Hsa-mir-184	Akt2^d^	Apoptosis↑, Invasiveness↓	[30]
Hsa-mir-185	DNMT1	DNA methylation↓	[124]
Hsa-mir-218	IKK-β^d^	Invasiveness↓	[25–27, 125]
Hsa-mir-326^bc^	Notch-1/2, PKM2^d^	Proliferation↓, Apoptosis↑, Viability↓, Invasiveness↓, In vivo tumor volume↓	[80, 81]
Hsa-mir-483-5p^b^	ERK1^d^	Proliferation↓	[24, 83]
Hsa-mir-491-5p^b^	MMP9^d^	Invasiveness↓	[37, 82]

miRNAs consistently downregulated in glioblastoma. The table presents miRNAs with observed effects upon their upregulation and their validated targets. The cell lines in which the studies have been performed are presented. Only miRNAs that are investigated in greater than or equal to five studies and/or have a validated target are included in this table. The cell lines applied in the annotated studies can be found in Supplementary Table 1

^a^Mentioned in the section “MicroRNAs downregulated in Glioblastoma Multiforme”

^b^Mentioned in the section “MicroRNAs Downregulated in Glioblastoma Multiforme with Limited Functional Characterization”

^c^MicroRNAs whose upregulation is shown to inhibit tumor growth in vivo

^d^Targets involved in the mesenchymal mode of migration and invasion

### miR-7

Seven studies published within the last 4 years have established miR-7 as being downregulated in GBM [25, 27, 31, 62–65]. A study by Lages et al. found a decrease in miR-7 expression of 0.11 and 0.09 fold in GBM and oligodendroglioma, respectively, suggesting that miR-7 also has a key role in tumors of non-astrocytic origin [31]. The first paper to functionally characterize miR-7 in GBM was published in 2008 by Kefas et al. and utilized a luciferase reporter assay to establish EGFR, frequently amplified in GBM [66], and IRS-2 as a direct targets of miR-7 [65]. Interestingly, the same paper found that transfection with miR-7 led to a decrease in phosphorylated Akt in a non EGFR-dependent manner and reduced invasiveness in several GBM cell lines [65]. More recently, it was shown that Focal Adhesion Kinase (FAK) is also a direct target of miR-7 and that overexpression of miR-7 resulted in decreased rates of both migration and invasion, possibly in part mediated by lowered levels MMP-2 and MMP-9 [63]. It is evident that miR-7 is an important suppressor of the MMMI, and future research could reveal further promising therapeutic characteristics.

### miR-34a

The first interesting study investigating miR-34a utilized both CD133+ and CD133− GBM cells, thus also examining the connection between the cancer stem cell hypothesis and miRNA. Here, Li et al. [50] found that forced overexpression of miR-34a resulted in inhibited proliferation and invasion both in vitro and in immunodeficient mice. It was found that these growth inhibitory effects on both CD133+ and CD133− cells were mediated by targeting the Notch signaling pathway via Notch1/2 and via c-Met [67]. Later, the group showed that miR-34a overexpression led to an increase in cell differentiation and apoptosis in a glioma stem cell culture [68]. The tumor-suppressive role of miR-34a was confirmed by Luan et al. who also found that miR-34a levels reflect the status of tumor suppressor p53, and that miR-34a could activate the p53 signaling cascade of p53 expression independently, possibly through targeting of SIRT1 [69]. Recently, two new targets of miR-34a were determined: Musashi1 and platelet-derived growth factor receptor-α (PDGFRA), last mentioned known to be amplified in GBM [70, 71]. Interestingly, a negative feedback mechanism between PDGFRA and miR-34a was discovered, implying that the underlying cause of miR-34a downregulation in GBM might be a result of increased PDGF-signaling [70]. Despite an obvious potential, intracranial delivery of miR-34a in the treatment of GBM has yet to be attempted.

### miR-128

Several papers have reported significant miR-128 repression in GBM cell lines and tumor samples [22, 24–26, 31, 35, 37, 52, 55, 71–75]. It is capable of suppressing tumor growth, mediated through numerous direct gene targets in a number of different cell lines. The first target investigated, Bmi-1, which normally drives stem cell renewal and glioma growth, was found to be downregulated upon miR-128 induction [75]. Adding to the antiproliferative effect of miR-128 is the fact that it mediates the silencing of the transcription factor E2F3a, an effect that was shown to be reversible by E2F3a restoration [73, 74]. Papagiannakopoulos et al. found the two growth factor receptors EGFR and PDGFRA, both typically overexpressed in GBM, to be repressed by miR-128 [76]. Other direct targets of miR-128 include WEE1 [25] and Msi1, involved in proliferation, and p70S6K1, involved in angiogenesis [72]. Based on the above-mentioned experimental evidence, it appears that miR-128 is a good candidate for repressing GBM growth and invasion.

### miR-137

Since its first description in relation to the pathology of GBM in 2008, miR-137 has been consistently reported as downregulated and has often been investigated in combination with other miRNAs, e.g., miR-124 [25–27, 35, 45, 52, 71, 77]. Studies have revealed antiproliferative and anti-invasive effects upon forced miR-137 overexpression, mediated through CDK6 [27], Msi1 [71], and Cox-2 [77]. Of significant interest here is the inhibition of Cox-2, as this enzyme is shown to play a role in cell proliferation [77].

### MicroRNAs Upregulated in Glioblastoma Multiforme with Limited Functional Characterization

As for the upregulated miRNAs, several specific downregulated miRNAs have been intensively investigated, while others, e.g., miR-32, miR-101, miR-326, and miR-491-5p, have received limited attention. Furthermore, mir-181b, mir-139-5p, mir-132, and mir-129 were found to be consistently downregulated in greater than or equal to five studies but remains uninvestigated with respect to functional role in GBM. miR-32 was found to be downregulated in three studies and shown to suppress intracranial xenograft tumor growth, thus improving overall survival rate of mice. Furthermore, two important p53 inhibitors, MDM2 and TSC1, were validated as direct targets of this miRNA [24, 45, 78]. Two studies found that miR-101 was able to decrease levels of angiogenesis, proliferation, migration, and viability by targeting part of the Polycomb-group family EZH2 and Msi1 [71, 79]. Lowered expression of EZH2 results in a reduction of histone methylation, affecting the expression of several genes involved in tumor suppression [71, 79].

Until now, only Kefas et al. [80, 81] have investigated miR-326 expression and function in GBM. In their two publications, they found that by targeting Notch1/2 and pyruvate kinase M2 (PKM2), miR-326 was able to decrease proliferation and invasiveness in a subset of different cell lines. In addition, they proposed that miR-326 and Notch1 regulate each other in a negative feedback manner [80, 81]. Another miRNA with anti-invasive properties is miR-491-5p, which is found to inhibit MMP9, a key component in the degradation of extracellular matrix [82].

The most recently published study on miR-483-5p has shown that it is decreased in both tumor samples and cell lines. Induction of miR-483-5p inhibited proliferation of GBM cells by arrest in the G0/G1 transition. Further verifying these results, was the interesting finding that miR-483-5p caused silencing of ERK1, known to be an important factor in several major mitogenic signaling pathways [83]. Clearly, the function of miR-483-5p is interesting with respect to its involvement in cell cycle arrest and its role in gene silencing of key regulatory pathways in tumorigenesis.

## MicroRNAs with Disputed Levels of Expression in Glioblastoma Multiforme

Besides the long list of up- and downregulated miRNAs are a group of conflicting experimental findings that need further resolution in order to define the key regulatory determinants. Throughout the literature review process, a number of studies could be identified where the expression levels of miRNAs were disputed (Table 3). In total, 17 miRNAs were found to be both up- and downregulated. Much of this could be attributed to the experimental framework of the studies, different models, and the well-known fact that the tumor niche is a governing factor with respect to gene regulation and expression [84, 85]. In the following section, a number of these disputed miRNAs will be discussed.

**Table 3:** miRNAs with disputed expression levels and their functional role in GBM

miRNA	Target	Functional role when overexpressed	Functional role when underexpressed	References	
				Overexpression in GBM	Underexpression in GBM
Hsa-mir-19a	CTGF		Viability↓, Apoptosis↑, Proliferation↓	[24–26, 30]	[34]
Hsa-mir-26b	EphA2^a^	Proliferation↓, Invasiveness↓, Angiogenesis↓		[31]	[26, 107]
Hsa-mir-27b	WEE1		Proliferation↓, Apoptosis↑, Invasiveness↓	[126]	[25]
Hsa-mir-106a	E2F1	Proliferation↓, Apoptosis↑		[24, 26, 35]	[45, 127]
Hsa-mir-143			Invasiveness↓	[6]	[26]
Hsa-mir-145^bc^	Oct4, SOX2	In vivo tumor volume↓, Migration↓, Stemness↓, Chemosensitivity↑, Radiosensitivity↑	Invasiveness↓	[6, 86]	[11]
Hsa-mir-205	VEGF-A^a^	Proliferation↓, Apoptosis↑, Invasiveness↓		[26]	[99]
Hsa-mir-451^b^	PI3K/Akt-pathway, CAB39^a^	Proliferation↓, Invasion↓, Stemness↓, Neurosphere formation↓, Proliferation↑	Migration↑	[23, 87, 89]	[35, 52, 88, 89]

miRNAs found to be both up- and downregulated in GBM and their functional role. The table presents miRNAs, which have been reported with differential expression levels in GBM between different research groups. Only miRNAs that are investigated in greater than or equal to five studies and/or have a validated target are included in this table. The effects of upregulation or downregulation and validated targets are presented. The cell lines applied in the annotated studies can be found in Supplementary Table 1

^a^Targets involved in the mesenchymal mode of migration and invasion

^b^Mentioned in the section “miRNAs with disputed expression levels”

^c^miRNAs whose upregulation is shown to inhibit tumor growth in vivo

### miR-145

One of the miRNAs with disputed findings in expression levels between nonmalignant brain tissue and GBM tumor samples/cell lines was miR-145 [6, 11, 86]. miR-145 was reported to be downregulated by Lee et al. [11] who also found a decrease in proliferation and invasion when inducing overexpression of miR-145. Furthermore, when inserting a miR-145 expression cassette into a HSVtk-expressing adenoviral vector, an increase in survival was observed following transmission to mice [11]. These observations were strengthened by Yang et al. [86] who showed increased radio- and chemosensitivity accompanied by a decrease in migration, stemness, and xenograft tumor growth upon miR-145 overexpression. These effects were found to be mediated through direct targeting of the two stemness transcription factors Oct4 and Sox2 [86]. However, contradictory to these two studies, Koo et al. [6] reported miR-145 to be upregulated in a number of highly invasive GBM cell lines, and that downregulation of miR-145 decreased the invasive abilities of GBM cells. Interestingly, the group also found miR-145 to be most upregulated in highly invasive regions of freshly resected human GBM tumor samples [6].

### miR-451

Generating another discrepancy in the literature are two papers exploring expression levels of miR-451 in GBM cell lines, finding dissimilar results [87, 88]. Gal et al. found that miR-451 was overexpressed in CD133− GBM cells, while Nan et al. found a downregulation of miR-451 in three GBM cell lines [87, 88]. However, both papers agreed that transfection with miR-451-mimics significantly decreased proliferation and viability, thus implying that miR-451 has tumor-suppressive effects in vitro. A possible explanation for these contradictory results might exist as presented by Godlewski et al. [89]. In two key papers, they investigated the relationship between metabolic stress in GBM cell lines and the expression of miR-451. Interestingly, they discovered that the cell migratory ability varies, dependent on glucose availability. Thus, when glucose levels are low, miR-451 is underexpressed, leading to increased migration via the AMPK/MAPK-pathway, mediated by direct targeting of calcium-binding protein 39 (CAB39) [90, 91].

## MicroRNAs and the Mesenchymal Mode of Migration and Invasion

Epithelial to mesenchymal transition (EMT) is a phenomenon during which epithelial cells lose many of their epithelial characteristics and acquire markers and phenotypes of mesenchymal cells. EMT facilitates metastasis through increased migration, invasion, intravasation and/or extravasation [92, 93]. Due to the astrocytic origin of GBM, a complementary concept called MMMI is proposed as a valuable notion in the malignant progression of glioblastoma (inspired by Zhong et al. [5]).

EMT encompasses many known pathways such as the EGFR, TGF-β, and Wnt-signaling that promote the continuous acquisition of malignant biological features by cancer cells and contributes to the highly invasive nature of certain cancers [94]. Essential transcription factors in EMT include ZEBs (delta-crystalline enhancer binding factors) which, together with the EMT-promoting transcription factor TWIST, regulate several of the EMT-associated pathways and enhance cellular invasion [95]. The ZEB and Twist proteins are implicated in EMT in several tumor types and regulate, or are under the regulation of miRNAs, underpinning that miRNAs are important regulators of malignancy development [93].

MMMI encompasses interaction between GBM cells and their surrounding extracellular matrix (ECM), through integrin-attachment and detachment resulting in FAK-signaling. This prompts several intracellular changes such as actin filamentation and myosin phosphorylation, which ultimately enables cell locomotion. Invasive GBM cells infiltrate the brain parenchyma and escape surgical resection and other local therapeutic modalities, and are considered a principle reason for tumor recurrence [5]. In the following section, the concept of MMMI is broadened to include all cellular processes, related to miRNAs, facilitating invasion and migration of GBM cells.

**Fig. 2:**
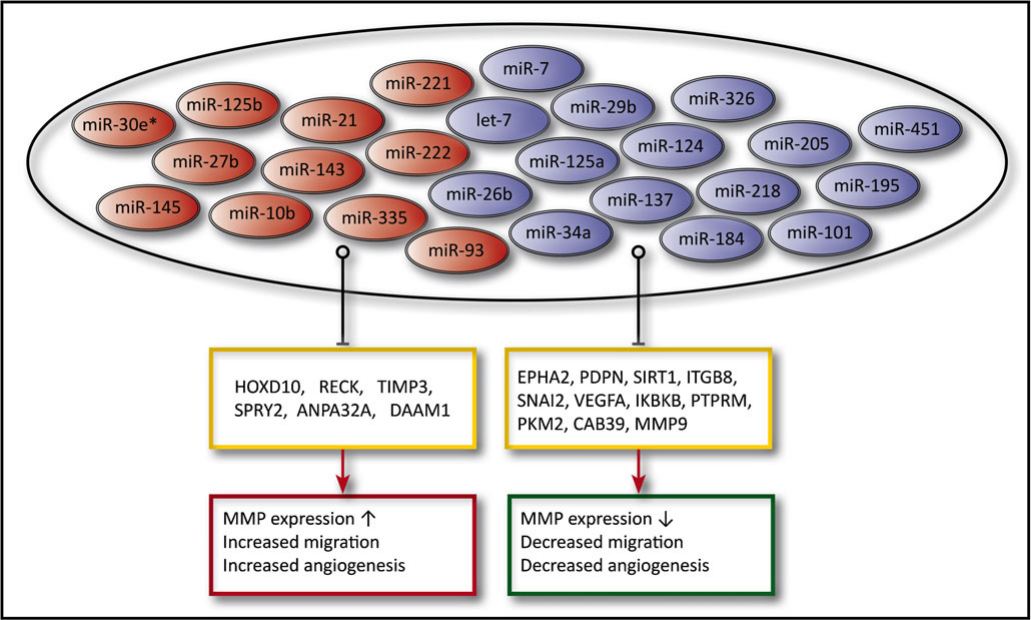
Schematic overview of miRNAs involved in the mesenchymal mode of migration and invasion in glioma. The inhibition of validated targets (*yellow boxes*) by specific miRNAs results in either pro-invasive (*red box*) or anti-invasive (*green box*) effects. *Red ovals* depict upregulated, oncogenic miRNAs, e.g., miR-10b targets HOXD10, resulting in increased MMP14 expression and invasion. *Blue ovals* depict downregulated tumor suppressor miRNAs in GBM, e.g., miR-26b targets EphA2, resulting in decreased levels of angiogenesis

It is clear from the literature that a number of miRNAs, both oncogenic and tumor suppressive, are involved in MMMI, thus affecting cell migration, cytoskeletal rearrangement, invasiveness, and angiogenesis (Fig. 2). This emphasizes the function of miRNAs as modulators of the ECM and tumor niche. miR-21, the most investigated miRNA in the literature, has been shown to be involved in many aspects regarding MMMI [22, 38, 93]. It is able to increase the expression and activity levels of a number of MMPs, facilitate Ras/Raf binding, and induce ERK phosphorylation through targeting of known tumor suppressors, like RECK, TIMP3, ANP32A, and SPRY2, thereby enhancing the invasive potential of the GBM cells [38, 39, 46, 48].

By inhibiting DAAM1, miR-335 is able to initiate cytoskeletal rearrangement and decrease the level of myosin light chain phosphorylation, which ultimately results in increased invasion [59]. Similar results have been obtained when exploring the effects of other known oncogenic miRNAs, such as miR-10b, miR-30e*, miR-125b, and miR-221 [29, 96–98].

In addition, 16 tumor-suppressive miRNAs (Fig. 2) are shown to play a role in the inhibition of invasion. For example, overexpression of miR-7, which targets EGFR and FAK, reduces the level of invasion in GBM, and diminishes the expressional levels of MMPs and phosphorylation of Akt and Erk [63–65]. miR-326 targets PKM2 with a resulting decrease in ATP levels, which is associated with less mTOR signaling and less invasion [81]. Furthermore, interesting results have been obtained regarding miR-491-5p, which directly targets MMP9 and, therefore, inhibits migration of GBM cells [82].

Another factor associated with invasion is angiogenesis, which is also under regulation of miRNAs. In addition to bringing nutrients to the tumor mass, angiogenesis is also required for facilitating tumor cell migration [93]. miRNAs, such as miR-93 and miR-205, have been shown to regulate the level of angiogenesis; miR-93 inhibits integrin-β8 with a resultant increase in angiogenesis [56], while miR-205, which targets VEGF-A, has the opposite effect [99]. Given the broad and very profound involvement of miRNAs in the regulation of gene expression and extracellular signaling, a focus on the miRNAs involved in MMMI and ECM dynamics as future agents, or targets for therapy, would be promising.

## Conclusion

In this comprehensive review of 102 papers, we have attempted to highlight the expression profile of miRNAs significantly up- or downregulated in GBM and subsequently focused on a group defined as more novel with respect to functional characterization. 253 miRNAs were found to be significantly upregulated, 95 significantly downregulated, and 17 were disputed with respect to expression levels###; 313 of these miRNAs have yet to be functionally characterized, prompting the need for further investigations. The function of miRNAs encompasses modulation of the ECM dynamics (Fig. 2) and likewise the miRNA expression can be influenced by the tumor niche [85]. Hence, the assembly of a group of miRNAs with disputed results in the levels of expression highlights the importance of tissue isolation/processing, choice of control tissue, and the tumor microenvironment; all of which are factors that could contribute to these differences in expression. Even though 365 miRNAs have been studied with respect to their expression in glioma, only 21 of them have been investigated with respect to their function in vivo (Supplementary Table 1). Furthermore, the 15 most studied miRNAs (miR-7, miR-10b, miR-15b, miR-17, miR-21, miR-23a, miR-25, miR-124, miR-128a, miR-128b, miR-132, miR-137, miR-195, miR-221, and miR-222) in GBM were investigated in 62 out of the 102 papers. The popularity per se of this cohort of well-investigated miRNAs sets a bias in the literature with respect to the importance of these miRNAs in glioma initiation, progression, and invasion. This leaves a large number of miRNAs that could hold a similar or greater potential in future therapeutics than the highly studied cohort.

## Electronic supplementary material

Below is the link to the electronic supplementary material.

ESM 1 (DOCX 478 kb)

## Footnotes

1 Search typed: “(microRNA AND Glioblastoma) OR (microRNA AND Glioma)”
