# Supplementary material for: A Systematic Review of MicroRNA in Glioblastoma Multiforme: Micro-modulators in the Mesenchymal Mode of Migration and Invasion
Source: Mol Neurobiol. 2012 Oct 2;47(1):131–44. doi: 10.1007/s12035-012-8349-7 (PMC3538124; doi:10.1007/s12035-012-8349-7)
Supplement: Supplementary file 1 — (DOCX 478 kb) [file 12035_2012_8349_MOESM1_ESM.docx]

| Supplement Table 1. Complete list of the miRNAs studied in glioblastoma | | | | | | |
| --- | --- | --- | --- | --- | --- | --- |
| miRNA | **Regulation in GBM** | **Targets** | **Overexpression** | **Underexpression** | **Cell line** | **Study** |
| **Hsa-Let-7** | Down |  | Migration↓, Proliferation↓, In vivo tumor volume↓ |  | U251, U87 | [24,127] |
| **Hsa-let-7a** | Up |  |  |  |  | [24] |
| **Hsa-let-7b** | Up |  |  |  |  | [24,28] |
| **Hsa-let-7c** | Up |  |  |  |  | [28] |
| **Hsa-let-7d** | Disputed |  |  |  |  | [12,24] |
| **Hsa-Let-7f** | Up |  |  |  |  | [24] |
| **Hsa-Let-7i** | Up |  |  |  |  | [28] |
| **Hsa-mir-1** | Down |  |  |  |  | [33,53] |
| **Hsa-mir-7** | Down | FAK, EGFR | Viability↓, Migration↓, Invasiveness↓, Proliferation↓, In vivo tumor volume↓, Radiosensitivity↓ |  | U87, U251, A172, T98, GBM10, GBM6 | [24,28,30,78-81] |
| **Hsa-mir-9** | Up | CAMTA1 |  |  |  | [23-27] |
| **Hsa-mir-9-2** | Up |  |  |  |  | [22] |
| **Hsa-mir-9*** | Up | CAMTA1 |  | Proliferation↓,  Stemness↓ |  | [26,28] |
| **Hsa-mir-10a** | Up |  |  |  |  | [25,27-29] |
| **Hsa-mir-10b** | Up | HOXD10 | Invasiveness↑ | Invasiveness↓ | U87, U251, U373, LN229, A172, T98G, YH13, SF126, U118 | [22,25,27-32] |
| **Hsa-mir-15a** | Up |  |  |  |  | [23,27,28,53] |
| **Hsa-mir-15b** | Up | CCNE1 | Proliferation↑ | Proliferation↓ |  | [24,25,27,28,33,34] |
| **Hsa-mir-16** | Up |  |  |  |  | [23,24,27,28,35] |
| **Hsa-mir-17-3p** | Up |  |  |  |  | [28] |
| **Hsa-mir-17** | Up | POLD2, TGFβ-RII, CTGF, CAMTA1 | Angiogenesis↑, Growth↑ | Viability↓, Apoptosis↑, Proliferation↓ | A172, T98G | [23,24,26-29,36,37] |
| **Hsa-mir-18a** | Up | Smad4, CTGF | Angiogenesis↑, Growth↑ | Viability↓, Apoptosis↑, Proliferation↓ | A172 | [28,36,37] |
| **Hsa-mir-18b** | Up |  |  |  |  | [28] |
| **Hsa-mir-19a** | Disputed | CTGF |  | Viability↓, Apoptosis↑, Proliferation↓ | A172 | [23,27-29,36,37] |
| **Hsa-mir-19b** | Disputed |  |  |  |  | [27-29,37] |
| **Hsa-mir-20a** | Up | TGFβ-RII, CTGF | Angiogenesis↑, Growth↑ | Viability↓, Proliferation↓ | A172 | [23,24,28,29,36] |
| **Hsa-mir-20b** | Up |  |  |  |  | [27,28] |
| **Hsa-mir-21** | Up | RECK, TIMP3, APAF1, ANP32A, SMARCA4, Caspases, PTEN, Cdc25A, HNRPK, TAp63, Spry2, LRRFIP1, PDCD4 | Invasiveness↑ | Invasiveness↓, Apoptosis↑, Viability↓, Proliferation↓, In vivo tumor volume↓, Chemosensitivity↑ | DBTRG-U5MG, U118, U87, A172, LN18, M059J, M059K, LN229, T98G, U138MG, U373, LN428, LN300, U251, SNB19, SF767 | [21-25,27-31,33,38-56,128] |
| **Hsa-mir-23a** | Up |  |  |  | DBTRG-U5MG, U118, U87, A172, LN18, M059J, M059K, LN229, T98G, U138MG | [22,25,27,28,33,38,53] |
| **Hsa-mir-23b** | Up |  |  |  | DBTRG-U5MG, U118, U87, A172, LN18, M059J, M059K, LN229, T98G, U138MG | [22,28,33,53] |
| **Hsa-mir-24** | Up |  |  |  |  | [28,46] |
| **Hsa-mir-24-1** | Up |  |  |  | DBTRG-U5MG, U118, U87, A172, LN18, M059J, M059K, LN229, T98G, U138MG | [22] |
| **Hsa-mir-24-2** | Up |  |  |  | DBTRG-U5MG, U118, U87, A172, LN18, M059J, M059K, LN229, T98G, U138MG | [22] |
| **Hsa-mir-25** | Up | Mdm2, TSC1 | In vivo tumor volume↓ |  |  | [22,23,25,27-29,57,83] |
| **Hsa-mir-26a** | Up | PTEN | In vivo tumor volume↑ |  |  | [25,28,38] |
| **Hsa-mir-26b** | Disputed | EphA2 | Proliferation↓, Invasiveness↓, Angiogenesis↓ |  | U251, U87, C6 | [24,29,66] |
| **Hsa-mir-27a** | Disputed | WEE1 |  |  |  | [25,28,53] |
| **Hsa-mir-27b** | Disputed | WEE1 | WEE1↓ | Proliferation↓, Apoptosis↑, Invasiveness↓ | TIC308, U87, LN229, SNB19, U251 | [28,114] |
| **Hsa-mir-28** | Up |  |  |  |  | [28] |
| **Hsa-mir-29b** | Down | PDPN | Invasiveness↓, Proliferation↓, Apoptosis↑ |  | U87, LN229, U251 | [28,35,46,82] |
| **Hsa-mir-29c** | Down |  |  |  |  | [28,35] |
| **Hsa-mir-30a-3p** | Up |  |  |  |  | [28] |
| **Hsa-mir-30a-p5** |  |  |  |  |  | [28] |
| **Hsa-mir-30a** | Up |  |  |  |  | [28] |
| **Hsa-mir-30c** | Disputed |  |  |  |  | [28,46] |
| **Hsa-mir-30d** | Up |  |  |  |  | [28] |
| **Hsa-mir-30e-3p** | Up |  |  |  |  | [28] |
| **Hsa-mir-30e** | Up | IκBα | Invasiveness↑, Proliferation↑, Angiogenesis↑, In vivo tumor volume↑ | Invasiveness↓, Proliferation↓, Angiogenesis↓, In vivo tumor volume↓ | U87, LN444, SNB19 | [28,58] |
| **Hsa-mir-32** | Down | Mdm2, TSC1 | In vivo tumor volume↓ |  |  | [27,46,83] |
| **Hsa-mir-33** | Down |  |  |  |  | [29,35] |
| **Hsa-mir-34a** | Down | SIRT1, c-Met, Notch1/2, PDGFRA, Msi1 | Viability↓, Proliferation↓  Apoptosis↑, Invasiveness↓, In vivo tumor volume↓, Differentiation↑ |  | SHG44, A172, U251, U87, TS543, TS667 | [84-88] |
| **Hsa-mir-92** | Up | CTGF |  | Viability↓ Proliferation↓ | A172 | [28,29,36,37] |
| **Hsa-mir-92b** | Up |  |  |  |  | [25,27,28,66] |
| **Hsa-mir-93** | Up | Integrin-β8 | Angiogenesis↑, Proliferation↑, In vivo tumor volume↑ |  | U87, U343 | [25,27,29,59] |
| **Hsa-mir-95** | Down |  |  |  |  | [12,33,53] |
| **Hsa-mir-96** | Up |  |  |  | LN18, U87, U118, T98G, SNB19, A172, U138, U251, U373 | [27,29,65] |
| **Hsa-mir-98** | Down |  |  |  |  | [29] |
| **Hsa-mir-99a** | Up |  |  |  |  | [27,28] |
| **Hsa-mir-99b** | Up |  |  |  |  | [28] |
| **Hsa-mir-100** | Down | ATM | Radiosensitivity↑ |  | M059K, M059J, U87 | [89] |
| **Hsa-mir-101** | Down | EZH2, Msi1 | Angiogenesis↓, Migration↓  Viability↓, Proliferation↓ |  | C6, U251, U87, U73, 293T, MCF-7, U118 | [88,129] |
| **Hsa-mir-103a** | Down |  |  |  |  | [12] |
| **Hsa-mir-103b** | Down |  |  |  |  | [12] |
| **Hsa-mir-105** | Up |  |  |  |  | [27] |
| **Hsa-mir-106a** | Disputed | E2F1 | Proliferation↓, Apoptosis↑ |  | U251, SHG44, U87, T98G, U373 | [27,29,33,46,115] |
| **Hsa-mir-106b** | Up |  |  |  |  | [25,27-29,31,53] |
| **Hsa-mir-107** | Down |  |  |  |  | [29,38] |
| **Hsa-mir-123** | Up |  |  |  |  | [22] |
| **Hsa-mir-124(a)** | Down | SNAI2 | Proliferation↓, Migration↓, Invasiveness↓, Stemness↓ |  | U251, SF126, U87, SF6969, A172, T98G, M059J, M059K, CCF-STGG | [12,27-30,33,35,38,46,53,91,92] |
| **Hsa-mir-124-1** | Down |  |  |  |  | [29] |
| **Hsa-mir-125a** | Down |  | Invasiveness↓ |  | U87, LN229, U251 | [46,82] |
| **Hsa-mir-125b** | Up | Bmf | Invasiveness↑, Apoptosis↓, Proliferation↑ |  | SU3, U251, U343 | [28,60-62] |
| **Hsa-mir-125b-1** | Up |  |  |  | DBTRG-U5MG, U118, U87, A172, LN18, M059J, M059K, LN229, T98G, U138MG | [22] |
| **Hsa-mir-125b-2** | Up |  |  |  | DBTRG-U5MG, U118, U87, A172, LN18, M059J, M059K, LN229, T98G, U138MG | [22] |
| **Hsa-mir-126** | Down |  |  |  |  | [29,33,53] |
| **Hsa-mir-127-3p** | Down |  |  |  |  | [24] |
| **Hsa-mir-127-5p** | Down |  |  |  |  | [33,53] |
| **Hsa-mir-128** | Down | WEE1, p70S6K1, Msi1, E2F3a, Bmi-1, EGFR, PDGFRα | Angiogenesis↓, Proliferation↓, In vivo tumor volume↓ |  | TIC308, U251, U87, T98G, A172 | [22,24,27-29,33,38,53,56,88,93-96] |
| **Hsa-mir-128b** | Down | WEE1 |  |  | DBTRG-U5MG, U118, U87, A172, LN18, M059J, M059K, LN229, T98G, U138MG, TIC308 | [12,22,28,29,33,38,53] |
| **Hsa-mir-129-3p** | Down |  |  |  |  | [27] |
| **Hsa-mir-129** | Down |  |  |  |  | [27-30,57] |
| **Hsa-mir-129*** | Down |  |  |  |  | [27] |
| **Hsa-mir-130a** | Up |  |  |  |  | [27-29] |
| **Hsa-mir-130b** | Up |  |  |  |  | [23,27-29] |
| **Hsa-mir-132** | Down |  |  |  |  | [12,24,27-29,33,53] |
| **Hsa-mir-133a** | Down |  |  |  |  | [28,29] |
| **Hsa-mir-133b** | Down |  |  |  |  | [29] |
| **Hsa-mir-134** | Down |  |  |  |  | [24,66] |
| **Hsa-mir-135a** | Down | STAT6, Smad5, BMPR2 |  | In vivo tumor volume↓, Apoptosis↑ | U87, C6, T98G, MGR2, U251 | [97] |
| **Hsa-mir-135b** | Up |  |  |  |  | [28,29] |
| **Hsa-mir-136** | Down |  |  |  |  | [29] |
| **Hsa-mir-137** | Down | CDK6, Msi1, Cox-2 | Proliferation↓, Invasiveness↓, Migration↓, In vivo tumor volume↓ |  | U87, SF6969, U251, LN229, SNB19, LN308, H4 | [28-30,33,46,53,88,98] |
| **Hsa-mir-138** | Down | Msi1 | Proliferation↓ |  | U251 | [27,38,88] |
| **Hsa-mir-138-1*** | Down |  |  |  |  | [12] |
| **Hsa-mir-139-3p** | Down |  |  |  |  | [12] |
| **Hsa-mir-139-5p** | Down |  |  |  |  | [12,24,28-30] |
| **Hsa-mir-140** | Up |  |  |  |  | [23] |
| **Hsa-mir-141** | Disputed |  |  |  |  | [28,29,38] |
| **Hsa-mir-142-3p** | Up |  |  |  |  | [25,27,38] |
| **Hsa-mir-142-5p** | Up |  |  |  |  | [25,27,28,57] |
| **Hsa-mir-143** | Disputed |  |  | Invasiveness↓ | U87, U251, U373, C6 | [6,29,38] |
| **Hsa-mir-144** | Up |  |  |  |  | [25,27,28] |
| **Hsa-mir-145** | Disputed | Oct4, SOX2 | In vivo tumor volume↓, Migration↓, Stemness↓, Chemosensitivity↑, Radiosensitivity↑ | Invasiveness↓ | U87, U251, U373, C6 | [6,116] |
| **Hsa-mir-146a** | Up | Notch1 | Proliferation↓, In vivo tumor volume↓, Migration↓ |  | U87 | [27,28,63] |
| **Hsa-mir-146b-5p** | Down | EGFR | Invasiveness↓, Migration↓, Proliferation↓, In vivo tumor volume↓ |  | U87, U251, HF66 | [99,130] |
| **Hsa-mir-147** | Up |  |  |  |  | [28] |
| **Hsa-mir-148a** | Up |  |  |  |  | [25] |
| **Hsa-mir-148b** | Up |  |  |  |  | [28] |
| **Hsa-mir-149** | Down | RAP1B, Wnt-pathway | Proliferation↓, Migration↓ |  | U251, SF126 | [24,38] |
| **Hsa-mir-150** | Up |  |  |  |  | [28] |
| **Hsa-mir-152** | Up |  |  |  |  | [27,28] |
| **Hsa-mir-153** | Down | Bcl-2, Mcl-1, Irs-2 | Proliferation↓, Viability↓, Apoptosis↑ |  | DBTRG-05MG, T698, LN18, U251 | [29,100,131] |
| **Hsa-mir-154** | Down |  |  |  |  | [29,46] |
| **Hsa-mir-155** | Up |  |  | Viability↓, Apoptosis↑, Chemosensitivity↑ | U251 | [24,27-29,35,64] |
| **Hsa-mir-181a** | Down | Bcl-2 | Proliferation↓, Apoptosis↑, Invasiveness↓, Radiosensitivity↑ |  | DBTRG-U5MG, U118, U87, A172, LN18, M059J, M059K, LN229, T98G, U138MG, U251, TJ905 | [22,38,101,102] |
| **Hsa-mir-181b** | Down |  | Proliferation↓, Apoptosis↑, Invasiveness↓ |  | DBTRG-U5MG, U118, U87, A172, LN18, M059J, M059K, LN229, T98G, U138MG, U251, TJ905 | [22,38,46,54,101] |
| **Hsa-mir-181c** | Down |  |  |  | DBTRG-U5MG, U118, U87, A172, LN18, M059J, M059K, LN229, T98G, U138MG | [22,56,101] |
| **Hsa-mir-181d** | Down | Bcl-2, K-Ras | Proliferation↓, Apoptosis↑, In vivo tumor volume↓ |  | U251, LN229 | [103] |
| **Hsa-mir-182** | Up |  |  |  | LN18, U87, U118, T98G, SNB19, A172, U138, U251, U373 | [29,38]}[25,27,28,65] |
| **Hsa-mir-182*** | Up |  |  |  |  | [28] |
| **Hsa-mir-183** | Up |  |  |  | LN18, U87, U118, T98G, SNB19, A172, U138, U251, U373 | [25,28,29,65,66] |
| **Hsa-mir-184** | Down | Akt2 | Apoptosis↑, Invasiveness↓ |  | A172, T98G | [23] |
| **Hsa-mir-185** | Down | DNMT1 | DNA methylation↓ |  | U251, U87, SF126, SF767 | [104] |
| **Hsa-mir-187*** | Down |  |  |  |  | [27] |
| **Hsa-mir-188** | Up |  |  |  |  | [29] |
| **Hsa-mir-189** | Up |  |  |  |  | [28] |
| **Hsa-mir-190** | Up |  |  |  |  | [28] |
| **Hsa-mir-191** | Up |  |  |  | DBTRG-U5MG, U118, U87, A172, LN18, M059J, M059K, LN229, T98G, U138MG | [22,28] |
| **Hsa-mir-191*** | Up |  |  |  |  | [28] |
| **Hsa-mir-192** | Up |  |  |  |  | [28] |
| **Hsa-mir-193** | Up |  |  |  |  | [28,29] |
| **Hsa-mir-193a-3p** | Up |  |  |  |  | [27] |
| **Hsa-mir-193b** | Up |  |  |  |  | [28] |
| **Hsa-mir-194** | Up |  |  |  |  | [28] |
| **Hsa-mir-195** | Disputed | CCND3, E2F3, CCND1 | Proliferation↓, Invasiveness↓ | Chemosensitivity, Viability↓ | U87, LN308, LN229, A172, T98G, U251, LN428, U373, U138 | [27,28,56,132-134] |
| **Hsa-mir-196a** | Up |  |  |  |  | [55,56] |
| **Hsa-mir-196b** | Up |  |  |  |  | [28,55,56] |
| **Hsa-mir-197** | Down |  |  |  | DBTRG-U5MG, U118, U87, A172, LN18, M059J, M059K, LN229, T98G, U138MG | [22] |
| **Hsa-mir-198** | Down |  |  |  |  | [27,28] |
| **Hsa-mir-199a-3p** | Up |  |  |  |  | [27,28] |
| **Hsa-mir-199a*** | Up |  |  |  |  | [28] |
| **Hsa-mir-199b-3p** | Up |  |  |  |  | [27] |
| **Hsa-mir-199b** | Up |  |  |  |  | [25,28] |
| **Hsa-mir-200a** | Up |  |  |  |  | [28] |
| **Hsa-mir-200b** | Up |  |  |  |  | [28] |
| **Hsa-mir-200c** | Up |  |  |  |  | [29] |
| **Hsa-mir-202** | Up |  |  |  |  | [28] |
| **Hsa-mir-203** | Down |  |  |  |  | [29] |
| **Hsa-mir-204** | Up |  |  |  |  | [28] |
| **Hsa-mir-205** | Disputed | VEGF-A | Proliferation↓, Apoptosis↑, Invasiveness↓ |  | H4, U87, LN229, U251 | [29] [117] |
| **Hsa-mir-206** | Up |  |  |  |  | [29] |
| **Hsa-mir-208** | Up |  |  |  |  | [28,29] |
| **Hsa-mir-210** | Up |  |  |  |  | [23,24,28,29,35] |
| **Hsa-mir-211** | Up |  |  |  |  | [28] |
| **Hsa-mir-212** | Up |  |  |  |  | [28] |
| **Hsa-mir-215** | Up |  |  |  |  | [28] |
| **Hsa-mir-216** | Up |  |  |  |  | [28,29] |
| **Hsa-mir-217** | Up |  |  |  |  | [28,29] |
| **Hsa-mir-218** | Down | IKK-β | Invasiveness↓ |  | U118, U373, U87, D247, SNB19, LN464, LN428, T98G, LN444 | [28-30,105] |
| **Hsa-mir-219** | Down |  |  |  |  | [27,33,46,53] |
| **Hsa-mir-219-2-3p** | Down |  |  |  |  | [27] |
| **Hsa-mir-220** | Up |  |  |  | DBTRG-U5MG, U118, U87, A172, LN18, M059J, M059K, LN229, T98G, U138MG | [22,28] |
| **Hsa-mir-221** | Up | P27, Akt, PUMA, P57, PTPμ | Proliferation↑, Invasiveness↑, In vivo tumor volume↑, Apoptosis↓, Migration↑ | Proliferation↓, Apoptosis↑, In vivo tumor volume↓ | DBTRG-U5MG, U118, U87, A172, LN18, M059J, M059K, LN229, T98G, U138MG, U251, H4, C6, LN308, LN319, LN228 | [22,33,53,54,67-73] |
| **Hsa-mir-222** | Up | Akt, PUMA, P57, PTPμ | Proliferation↑, Invasiveness↑, In vivo tumor volume↑, Apoptosis↓, Migration↑ | Proliferation↓, Apoptosis↑, in vivo tumor volume↓ | DBTRG-U5MG, U118, U87, A172, LN18, M059J, M059K, LN229, T98G, U138MG, U251, H4, C6, LN308, LN319, LN228 | [22,33,53,68-73] |
| **Hsa-mir-223** | Disputed |  |  |  |  | [25,33,53] |
| **Hsa-mir-224** | Up |  |  |  |  | [28] |
| **Hsa-mir-296** | Up |  |  |  |  | [28,29] |
| **Hsa-mir-299** | Down |  |  |  |  | [29] |
| **Hsa-mir-301a** | Up |  |  |  |  | [28,29] |
| **Hsa-mir-302a** | Up |  |  |  |  | [28,29] |
| **Hsa-mir-302b** | Disputed |  |  |  |  | [28,29,38] |
| **Hsa-mir-302c** | Disputed |  |  |  |  | [28,29,31] |
| **Hsa-mir-302d** | Up |  |  |  |  | [29] |
| **Hsa-mir-320a** | Up |  |  |  |  | [27-29] |
| **Hsa-mir-323** | Down |  |  |  |  | [29,33,53] |
| **Hsa-mir-323-3p** | Down |  |  |  |  | [12,27] |
| **Hsa-mir-323b-3p** | Down |  |  |  |  | [12] |
| **Hsa-mir-324-3p** | Up |  |  |  |  | [28] |
| **Hsa-mir-324-5p** | Up |  |  |  |  | [38] |
| **Hsa-mir-325** | Up |  |  |  |  | [28] |
| **Hsa-mir-326** | Down | Notch 1/2, PKM2 | Proliferation↓, Apoptosis↑, Viability↓, Invasiveness↓, In vivo tumor volume↓ |  | U87, U251, T98G, U373, A172 | [106,107] |
| **Hsa-mir-328** | Down |  |  |  |  | [23,29] |
| **Hsa-mir-329** | Down |  |  |  |  | [27,31,33,53] |
| **Hsa-mir-330-3p** | Down |  |  |  |  | [24,33,53] |
| **Hsa-mir-330-5p** | Up |  |  |  |  | [28] |
| **Hsa-mir-335** | Up | Daam1 | Viability↑, Invasiveness↑ | Apoptosis↑, Invasiveness↓, In vivo tumor volume↓ |  | [28,74] |
| **Hsa-mir-337** | Up |  |  |  |  | [28] |
| **Hsa-mir-338-3p** | Down |  |  |  |  | [27-29,53] |
| **Hsa-mir-338-5p** | Disputed |  |  |  |  | [27,28,38] |
| **Hsa-mir-339-5p** | Down |  |  |  |  | [24] |
| **Hsa-mir-340** | Up |  |  |  |  | [28] |
| **Hsa-mir-342** | Down |  |  |  |  | [33,53] |
| **Hsa-mir-345** | Up |  |  |  |  | [28] |
| **Hsa-mir-361** | Up |  |  |  |  | [28] |
| **Hsa-mir-362-3p** | Up |  |  |  |  | [27] |
| **Hsa-mir-362** | Up |  |  |  |  | [25,28] |
| **Hsa-mir-363** | Up |  |  |  |  | [28] |
| **Hsa-mir-363*** | Up |  |  |  |  | [28] |
| **Hsa-mir-367** | Up |  |  |  |  | [28,29] |
| **Hsa-mir-369** | Down |  |  |  |  | [29,31] |
| **Hsa-mir-370** | Down |  |  |  |  | [29] |
| **Hsa-mir-371** | Up |  |  |  |  | [28,29] |
| **Hsa-mir-372** | Up |  |  |  |  | [29] |
| **Hsa-mir-373** | Up |  |  |  |  | [28,29] |
| **Hsa-mir-374a** | Up |  |  |  |  | [24,27] |
| **Hsa-mir-374b** | Up |  |  |  |  | [27] |
| **Hsa-mir-375** | Up |  |  |  |  | [28] |
| **Hsa-mir-376a** | Down |  |  |  |  | [38] |
| **Hsa-mir-376b** | Up |  |  |  |  | [28] |
| **Hsa-mir-377** | Up |  |  |  |  | [28] |
| **Hsa-mir-378** | Up |  |  |  |  | [28] |
| **Hsa-mir-379** | Down |  |  |  |  | [28,31] |
| **Hsa-mir-380-3p** | Up |  |  |  |  | [28] |
| **Hsa-mir-380-5p** | Up |  |  |  |  | [28] |
| **Hsa-mir-381** | Up | LRRC4 | Proliferation↑, In vivo tumor volume↑ | Proliferation↓ | U87, U251, P19, SF126, SF767 | [75] |
| **Hsa-mir-383** | Up |  |  |  |  | [28] |
| **Hsa-mir-384** | Up |  |  |  |  | [28] |
| **Hsa-mir-409-5p** | Down |  |  |  |  | [24] |
| **Hsa-mir-410** | Down |  |  |  |  | [28] |
| **Hsa-mir-411** | Up |  |  |  |  | [28] |
| **Hsa-mir-421** | Up |  |  |  |  | [28] |
| **Hsa-mir-422a** | Down |  |  |  |  | [28] |
| **Hsa-mir-423** | Up |  |  |  |  | [28] |
| **Hsa-mir-424** | Up |  |  |  |  | [27] |
| **Hsa-mir-425-5p** | Up |  |  |  |  | [28] |
| **Hsa-mir-429** | Up |  |  |  |  | [28] |
| **Hsa-mir-431** | Up |  |  |  |  | [28] |
| **Hsa-mir-432** | Down |  |  |  |  | [27] |
| **Hsa-mir-450a** | Up |  |  |  |  | [27] |
| **Hsa-mir-451** | Disputed | PI3K/Akt-pathway, CAB39 | Proliferation↓Invasion↓, Stemness↓, Neurosphere formation↓, Proliferation↑ | Migration↑ | U251, A172, LN229, U87, U373 | [25,33,53,118-120] |
| **Hsa-mir-452** | Up |  |  |  |  | [28] |
| **Hsa-mir-452*** | Up |  |  |  |  | [28] |
| **Hsa-mir-454-3p** | Down |  |  |  |  | [28] |
| **Hsa-mir-454-5p** | Down |  |  |  |  | [28] |
| **Hsa-mir-455** | Up |  |  |  |  | [28] |
| **Hsa-mir-483-5p** | Down | ERK1 | Proliferation↓ |  | U251, U87, SHG44 | [27,108] |
| **Hsa-mir-484** | Up |  |  |  |  | [28] |
| **Hsa-mir-486** | Up |  |  |  |  | [25] |
| **Hsa-mir-487a** | Down |  |  |  |  | [28] |
| **Hsa-mir-487b** | Down |  |  |  |  | [12,28] |
| **Hsa-mir-491-5p** | Down | MMP9 | Invasiveness↓ |  | U87, U251 | [38,109] |
| **Hsa-mir-493-3p** | Up |  |  |  |  | [28] |
| **Hsa-mir-493-5p** | Up |  |  |  |  | [28] |
| **Hsa-mir-495** | Down |  |  |  |  | [28,33,53] |
| **Hsa-mir-497** | Up |  |  |  |  | [28] |
| **Hsa-mir-499** | Up |  |  |  |  | [28] |
| **Hsa-mir-500** | Up |  |  |  |  | [28] |
| **Hsa-mir-501** | Up |  |  |  |  | [28] |
| **Hsa-mir-503** | Up |  |  |  |  | [25,28] |
| **Hsa-mir-505** | Up |  |  |  |  | [25,27] |
| **Hsa-mir-506** | Up |  |  |  |  | [28] |
| **Hsa-mir-507** | Up |  |  |  |  | [28] |
| **Hsa-mir-508** | Up |  |  |  |  | [28] |
| **Hsa-mir-509** | Up |  |  |  |  | [28] |
| **Hsa-mir-511** | Up |  |  |  |  | [28] |
| **Hsa-mir-512-5p** | Up |  |  |  |  | [28] |
| **Hsa-mir-513** | Up |  |  |  |  | [28] |
| **Hsa-mir-514** | Up |  |  |  |  | [28] |
| **Hsa-mir-515-5p** | Up |  |  |  |  | [28] |
| **Hsa-mir-516-3p** | Up |  |  |  |  | [28] |
| **Hsa-mir-517*** | Up |  |  |  |  | [28] |
| **Hsa-mir-517c** | Up |  |  |  |  | [28] |
| **Hsa-mir-518b** | Up |  |  |  |  | [28] |
| **Hsa-mir-518c** | Up |  |  |  |  | [28] |
| **Hsa-mir-518d** | Up |  |  |  |  | [28] |
| **Hsa-mir-518f** | Up |  |  |  |  | [28] |
| **Hsa-mir-519a** | Up |  |  |  |  | [28] |
| **Hsa-mir-519b** | Up |  |  |  |  | [28] |
| **Hsa-mir-519c** | Up |  |  |  |  | [28] |
| **Hsa-mir-519d** | Up |  |  |  |  | [28] |
| **Hsa-mir-520c** | Up |  |  |  |  | [28] |
| **Hsa-mir-520d** | Up |  |  |  |  | [28] |
| **Hsa-mir-520f** | Up |  |  |  |  | [28] |
| **Hsa-mir-521** | Up |  |  |  |  | [28] |
| **Hsa-mir-523** | Up |  |  |  |  | [28] |
| **Hsa-mir-524** | Up |  |  |  |  | [28] |
| **Hsa-mir-524*** | Up |  |  |  |  | [28] |
| **Hsa-mir-525** | Up |  |  |  |  | [28] |
| **Hsa-mir-526a** | Up |  |  |  |  | [28] |
| **Hsa-mir-526c** | Up |  |  |  |  | [28] |
| **Hsa-mir-527** | Up |  |  |  |  | [28] |
| **Hsa-mir-532** | Up |  |  |  |  | [25,28] |
| **Hsa-mir-542-3p** | Up |  |  |  |  | [25,28] |
| **Hsa-mir-542-5p** | Up |  |  |  |  | [25,27,28] |
| **Hsa-mir-543** | Down |  |  |  |  | [12] |
| **Hsa-mir-544** | Up |  |  |  |  | [28] |
| **Hsa-mir-548b** | Up |  |  |  |  | [28] |
| **Hsa-mir-548c** | Up |  |  |  |  | [28] |
| **Hsa-mir-550** | Up |  |  |  |  | [25,27,28] |
| **Hsa-mir-551b** | Up |  |  |  |  | [28] |
| **Hsa-mir-552** | Up |  |  |  |  | [28] |
| **Hsa-mir-553** | Up |  |  |  |  | [28] |
| **Hsa-mir-554** | Up |  |  |  |  | [28] |
| **Hsa-mir-555** | Up |  |  |  |  | [28] |
| **Hsa-mir-559** | Up |  |  |  |  | [28] |
| **Hsa-mir-561** | Up |  |  |  |  | [28] |
| **Hsa-mir-562** | Up |  |  |  |  | [28] |
| **Hsa-mir-563** | Up |  |  |  |  | [28] |
| **Hsa-mir-564** | Up |  |  |  |  | [28] |
| **Hsa-mir-565** | Up |  |  |  |  | [28] |
| **Hsa-mir-566** | Up |  |  |  |  | [28] |
| **Hsa-mir-567** | Up |  |  |  |  | [28] |
| **Hsa-mir-570** | Up |  |  |  |  | [28] |
| **Hsa-mir-572** | Up |  |  |  |  | [28] |
| **Hsa-mir-574** | Down |  |  |  |  | [28] |
| **Hsa-mir-576** | Up |  |  |  |  | [28] |
| **Hsa-mir-577** | Up |  |  |  |  | [28] |
| **Hsa-mir-584** | Down |  |  |  |  | [28] |
| **Hsa-mir-585** | Up |  |  |  |  | [38] |
| **Hsa-mir-587** | Up |  |  |  |  | [28] |
| **Hsa-mir-588** | Up |  |  |  |  | [28] |
| **Hsa-mir-589** | Up |  |  |  |  | [28] |
| **Hsa-mir-591** | Up |  |  |  |  | [28] |
| **Hsa-mir-593** | Up |  |  |  |  | [28] |
| **Hsa-mir-594** | Up |  |  |  |  | [28] |
| **Hsa-mir-596** | Up |  |  |  |  | [28] |
| **Hsa-mir-597** | Up |  |  |  |  | [28] |
| **Hsa-mir-598** | Down |  |  |  |  | [12] |
| **Hsa-mir-599** | Up |  |  |  |  | [28] |
| **Hsa-mir-600** | Up |  |  |  |  | [28] |
| **Hsa-mir-602** | Up |  |  |  |  | [28] |
| **Hsa-mir-604** | Up |  |  |  |  | [28] |
| **Hsa-mir-609** | Up |  |  |  |  | [28] |
| **Hsa-mir-612** | Up |  |  |  |  | [28] |
| **Hsa-mir-616** | Up |  |  |  |  | [28] |
| **Hsa-mir-617** | Up |  |  |  |  | [28] |
| **Hsa-mir-619** | Up |  |  |  |  | [28] |
| **Hsa-mir-620** | Up |  |  |  |  | [28] |
| **Hsa-mir-622** | Up |  |  |  |  | [28] |
| **Hsa-mir-623** | Up |  |  |  |  | [28] |
| **Hsa-mir-624** | Up |  |  |  |  | [28] |
| **Hsa-mir-625** | Up |  |  |  |  | [28] |
| **Hsa-mir-627** | Up |  |  |  |  | [28] |
| **Hsa-mir-628-3p** | Down |  |  |  |  | [27] |
| **Hsa-mir-629** | Down |  |  |  |  | [28] |
| **Hsa-mir-630** | Up |  |  |  |  | [28] |
| **Hsa-mir-635** | Up |  |  |  |  | [28] |
| **Hsa-mir-636** | Up |  |  |  |  | [28] |
| **Hsa-mir-637** | Down |  |  |  |  | [27] |
| **Hsa-mir-638** | Down |  |  |  |  | [27] |
| **Hsa-mir-639** | Up |  |  |  |  | [28] |
| **Hsa-mir-643** | Up |  |  |  |  | [28] |
| **Hsa-mir-646** | Up |  |  |  |  | [28] |
| **Hsa-mir-648** | Up |  |  |  |  | [28] |
| **Hsa-mir-649** | Up |  |  |  |  | [28] |
| **Hsa-mir-651** | Up |  |  |  |  | [28] |
| **Hsa-mir-652** | Up |  |  |  |  | [28] |
| **Hsa-mir-653** | Up |  |  |  |  | [28] |
| **Hsa-mir-654** | Up |  |  |  |  | [28] |
| **Hsa-mir-658** | Up |  |  |  |  | [28] |
| **Hsa-mir-661** | Up |  |  |  |  | [28] |
| **Hsa-mir-662** | Up |  |  |  |  | [28] |
| **Hsa-mir-663** | Up |  |  |  |  | [38] |
| **Hsa-mir-665** | Down |  |  |  |  | [27] |
| **Hsa-mir-668** | Up |  |  |  |  | [28] |
| **Hsa-mir-765** | Down |  |  |  |  | [27] |
| **Hsa-mir-766** | Up |  |  |  |  | [28] |
| **Hsa-mir-768-3p** | Up |  |  |  |  | [28] |
| **Hsa-mir-802** | Up |  |  |  |  | [28] |
| **Hsa-mir-873** | Down |  |  |  |  | [12] |
| **Hsa-mir-885-5p** | Up |  | Invasiveness↓ |  | U87, U251 | [109] |
| **Hsa-mir-886-3p** | Up |  |  |  |  | [38] |
| **Hsa-mir-886-5p** | Up |  |  |  |  | [38] |
| **Hsa-mir-1908** | Down |  |  |  |  | [27] |
| **Hsa-mirPlus-A1027** | Down |  |  |  |  | [27] |
| **Hsa-mirPlus-A1056** | Down |  |  |  |  | [27] |
| **Hsa-mirPlus-D1036** | Down |  |  |  |  | [27] |
| Supplement table 1. The table presents all miRNAs that until now have been investigated with respect to expression and/or function in glioblastoma. Included is validated gene targets, effects upon up- or downregulation of the particular miRNA and the cell lines in which this functional characterization has been explored. | | | | | | |

**References**

These references are only cited in the supplemental material.

128. Lee S-T, Chu K, Oh H-J, Im W-S, Lim J-Y, Kim S-K, Park C-K, Jung K-H, Lee SK, Kim M, Roh J-K (2011) Let-7 microRNA inhibits the proliferation of human glioblastoma cells. J Neurooncol 102(1):19–24

129. Smits M, Mir SE, Nilsson RJA, van der Stoop PM, Niers JM, Marquez VE, Cloos J, Breakefield XO, Krichevsky AM, Noske DP, Tannous BA, Würdinger T (2011) Down-regulation of miR-101 in endothelial cells promotes blood vessel formation through reduced repression of EZH2. PLoS One 6(1):e16282

130. Xia H, Qi Y, Ng SS, Chen X, Li D, Chen S, Ge R, Jiang S, Li G, Chen Y, He M-L, Kung H-F, Lai L, Lin MC (2009) microRNA-146b inhibits glioma cell migration and invasion by targeting MMPs. Brain Res 1269:158–165

131. Xu J, Liao X, Lu N, Liu W, Wong C-W (2011) Chromatin-modifying drugs induce miRNA-153 expression to suppress Irs-2 in glioblastoma cell lines. Int J Cancer 129(10):2527–2531

132. Zhang Q-Q, Xu H, Huang M-B, Ma L-M, Huang Q-J, Yao Q, Zhou H, Qu L-H (2012) MicroRNA-195 plays a tumor-suppressor role in human glioblastoma cells by targeting signaling pathways involved in cellular proliferation and invasion. Neuro Oncol 14(3):278–287

133. Biosystems A (2005) MicroRNA expression signature in human glioblastoma multiforme brain tumor. Appl Biosyst. p. Poster

134. Ujifuku K, Mitsutake N, Takakura S, Matsuse M, Saenko V, Suzuki K, Hayashi K, Matsuo T, Kamada K, Nagata I, Yamashita S (2010) miR-195, miR-455-3p and miR-10a( *) are implicated in acquired temozolomide resistance in glioblastoma multiforme cells. Cancer Lett 296(2):241–248
